# Structural changes in lignocellulosic biomass during activation with ionic liquids comprising 3-methylimidazolium cations and carboxylate anions

**DOI:** 10.1186/s13068-018-1263-0

**Published:** 2018-09-27

**Authors:** Preenaa Moyer, Keonhee Kim, Nourredine Abdoulmoumine, Stephen C. Chmely, Brian K. Long, Danielle Julie Carrier, Nicole Labbé

**Affiliations:** 1 Center for Renewable Carbon, University of Tennessee, Knoxville, TN 37996 USA; 2 Department of Biosystems Engineering and Soil Science, University of Tennessee, Knoxville, TN 37996 USA; 3 Department of Chemistry, University of Tennessee, Knoxville, TN 37996 USA

**Keywords:** Ionic liquids, Lignocellulosic biomass, Pretreatment, Activation, Low severity, Fractionation, [EMIM][CH_3_COO], [AMIM][HCOO], Acetate, Formate

## Abstract

**Background:**

Lignocellulosic biomass requires either pretreatment and/or fractionation to recover its individual components for further use as intermediate building blocks for producing fuels, chemicals, and products. Numerous ionic liquids (ILs) have been investigated for biomass pretreatment or fractionation due to their ability to activate lignocellulosic biomass, thereby reducing biomass recalcitrance with minimal impact on its structural components. In this work, we studied and compared 1-allyl-3-methylimidazolium formate ([AMIM][HCOO]) to the commonly used 1-ethyl-3-methylimidazolium acetate ([EMIM][CH_3_COO]) for its potential to activate hybrid poplar biomass and enable high cellulose and hemicellulose enzymatic conversion. Although [EMIM][CH_3_COO] has been widely used for activation, [AMIM][HCOO] was recently identified to achieve higher biomass solubility, with an increase of 40% over [EMIM][CH_3_COO].

**Results:**

Since IL activation is essentially an early stage of IL dissolution, we assessed the recalcitrance of [EMIM][CH_3_COO] and [AMIM][HCOO]-activated biomass through a suite of analytical tools. More specifically, Fourier transform infrared spectroscopy and X-ray diffraction showed that activation using [AMIM][HCOO] does not deacetylate hybrid poplar as readily as [EMIM][CH_3_COO] and preserves the crystallinity of the cellulose fraction, respectively. This was supported by scanning electron microscopy and enzymatic saccharification experiments in which [EMIM][CH_3_COO]-activated biomass yielded almost twice the cellulose and hemicellulose conversion as compared to [AMIM][HCOO]-activated biomass.

**Conclusion:**

We conclude that the IL [AMIM][HCOO] is better suited for biomass dissolution and direct product formation, whereas [EMIM][CH_3_COO] remains the better IL for biomass activation and fractionation.

## Background

Current fossil fuel consumption emits an alarming quantity of carbon dioxide into the atmosphere and is often associated to the rise in average daily temperatures. In September 2017, NASA’s Goddard Institute for Space Studies (GISS) stated that the surface temperatures in 2017 were consistent with the global average trends observing a gradually warmer climate since 1980 [1]. Therefore, there is an urgent need for alternatives to produce chemicals and fuels from a renewable and sustainable carbon source such as lignocellulosic biomass.

Lignocellulosic biomass is mainly comprised of cellulose (30–45%), hemicellulose (20–40%), and lignin (5–35%) [2–4]. Cellulose is made of d-glucose monomer units linked by β (1 → 4) glycosidic bonds and is a highly stable linear homopolymer, unlike hemicellulose and lignin, which have random and less ordered structures. Hemicellulose is made of a diverse classes of polysaccharides, including xylan, glucuronoxylan, arabinoxylan, glucomannan, and xyloglucan [5]. Lignin reinforces the cell wall of plants and forms a physical barrier against chemical, biological, or physical attacks. Altogether, the heterogeneous structure and complexity of cell wall constituents are the main contributors to biomass recalcitrance [6]. This recalcitrant structure of biomass hinders its conversion into various streams that can then be transformed into fuels and other chemicals and products. For biorefinery applications to be cost-effective, an efficient biomass pretreatment or fractionation method is imperative for maximizing conversion into intermediate products.

The recent need for biomass valorization requires biorefineries to optimize conversion of lignocellulosic biomass, i.e., cellulose and hemicellulose into soluble sugars, and lignin into a high purity fraction that can be transformed into other chemical products [7–9]. Pretreatment methods such as dilute acid treatment, autohydrolysis, steam explosion, wet oxidation, and ammonia fiber expansion (AFEX) have not been designed to recover all three components [10, 11]. Pretreatment processes typically target either cellulose or/and hemicellulose, but degrade the lignin fraction. In contrast, fractionation processes based on the use of solvents such as ethanol, acetone, γ-valerolactone (GVL), tetrahydrofuran (THF), and ionic liquids are useful for recovering all three components [12–14]. As a fractionation process, organosolv (which uses an organic solvent often along with an acid catalyst) is known to produce lignocellulosic fractions with high purity with a recovered lignin that is partially depolymerized and does not contain new carbon–carbon bonds [15]. However, volatile solvents, high temperatures, and pressures make this process hazardous, requiring special expensive reaction vessels [16]. Biomass fractionation approaches using GVL and THF have been recently developed and these are still being studied for their potential as a pathway for complete separation and recovery of biomass components [17, 18].

Ionic liquids (ILs) are salts with low melting points and high vapor pressure. They are typically non-toxic and many are suitable for biomass pretreatment, which is commonly carried out at temperatures over 100 °C and with residence times ranging from 30 min to several days [19–22]. Ionic liquids are the only solvents that can be used for biomass pretreatment, fractionation, and dissolution. During IL pretreatment, a cellulose-rich fraction can be generated through the degradation and removal of a large portion of lignin and hemicellulose [23]. Conversely, biomass fractionation involves an IL activation step, which employs mild conditions, resulting in the “loosening” of the plant cell wall, a term coined to indicate the weakening of cell wall linkages [8, 24]. Finally, IL dissolution describes complete or partial solubilization of biomass in ILs for direct product formation as opposed to activation and pretreatment processes which produce intermediate products (sugars and lignin). Although the terms activation and pretreatment are used interchangeably, these are not similar as activation is used to describe processes that are carried out at milder reaction temperatures of 50–80 °C with the main of goal of recovering all the biomass constituents and minimizing their degradation [25]. After activation, biomass can be directly subjected to in situ enzymatic saccharification or regenerated through a solute displacement mechanism using an anti-solvent for ex situ saccharification [26]. The IL activation step allows the saccharide fraction, including hemicellulose and cellulose, to become less intertwined with lignin and undergo maximum hydrolysis to glucose during enzymatic saccharification [22, 27]. Lignin can then be recovered in the solid fraction.

One of the main advantages of IL activation is the cleavage of acetyl groups in hemicellulose and the decrease in cellulose crystallinity, resulting in significant changes in the overall biomass properties [27]. Unlike hemicellulose and lignin, cellulose has a crystalline structure; the reduction of the crystallinity of cellulose is critical for providing access to hydrolytic enzymes [28]. Due to the mild processing conditions used in IL activation, structural changes that occur within the biomass do not compromise chemical composition, as is encountered with the use of more traditional pretreatment technologies (dilute acid pretreatment, alkali pretreatment, autohydrolysis, and AFEX). During IL activation and fractionation, total biomass carbon content is mostly preserved, apart from the carbon loss due to deacetylation. A widely used IL for both pretreatment and activation is 1-ethyl-3-methylimidazolium acetate or [EMIM][CH_3_COO] [12, 29, 30]. While other ILs, such as 1-allyl-3-methylimidazolium chloride ([AMIM][Cl]) and 1-butyl-3-methylimidazolium chloride ([BMIM][Cl]), have been used for biomass pretreatment or activation, [EMIM][CH_3_COO] is known to be an effective cellulose solvent, inducing changes in crystallinity even during IL activation.

As a pretreatment solvent, [EMIM][CH_3_COO] has been reported to be highly efficient at a temperature of 140 °C and a pretreatment time of 3 h for maximum production of sugars from switchgrass [22]. In another study using biphasic and triphasic systems with processing times of 44 h and temperature of 70 °C, [EMIM][CH_3_COO] pretreatment resulted in 100% cellulose conversion for miscanthus grass [21]. For activation/fractionation, yellow poplar biomass activated and regenerated with [EMIM][CH_3_COO] for 72 h at 60 °C with a 4 wt% biomass loading resulted in about 100% conversion of the cellulose fraction into glucose within 48 h [27]. Switchgrass activated with tetrabutylammonium hydroxide ([TBA][OH]) at 50 °C for 3 h at a 20 wt% biomass loading resulted in glucose yields of ~ 95% [25]. The previous work using [TBA][OH] by the US Department of Energy’s Joint BioEnergy Institute (JBEI) highlighted the use of a higher biomass loading during pretreatment with this IL, which is essential for enhancing energy efficiency and cost-effectiveness of biorefinery processes [25]. In addition, ILs used for pretreatment as well as fractionation can be recycled in a closed loop system [22]. Shill et al. [21] described a process in which the IL [EMIM][CH_3_COO] was recycled after biomass pretreatment upon adding an aqueous solution containing a kosmotropic anion. The precipitation of cellulose from the IL allowed for its recovery, purification, and recycle. Despite all the progress made, there remains a fundamental gap in peer-reviewed literature, in that the use of IL activation for the simultaneous conversion of cellulose and recovery of lignin has not been systematically studied.

Optimizing IL activation to convert cellulose and hemicellulose into soluble sugars and recover high purity lignin requires lower severity conditions (i.e., shorter reaction times and lower reaction temperatures), while still using a high biomass loading. Performing tests under conditions required for complete dissolution/solubilization of biomass in an IL is a good indicator of the maximum biomass loading that can be achieved at specific time and temperature ranges during IL activation. Biomass solubility in ILs is highly dependent on each lignocellulosic component. While lignin and cellulose are known to readily dissolve in common ILs, dissolution of hemicellulose in [EMIM][CH_3_COO] only reaches approximately 5 wt% under similar conditions and concentrations [31]. This is often caused by the hemicellulose coating of cellulosic fibrils that remain intact during activation, requiring treatment for either longer times or higher temperatures. Hemicellulose is a critical barrier to cellulose conversion and its partial removal results in the cleavage of lignin carbohydrate complexes (LCC), which disrupts linkages between lignin and hemicellulose and allows for higher sugar yields [32, 33]. More recent works by Deb et al. [34] has shown that partial removal of hemicellulose, using a coupled autohydrolysis step, allowed birch saw dust to completely dissolve in [AMIM][Cl] within 3 h. Similarly, Wang et al. [35] partially removed 55% of hemicellulose from switchgrass, resulting in an [EMIM][CH_3_COO] activation time of only 3 h to completely convert cellulose into glucose. Wang et al. [36] also showed that the partial removal of the hemicellulose was required to enhance biomass solvation in [EMIM][CH_3_COO], achieving optimal biomass loading and solution viscosity for the direct production of lignocellulosic films. These studies lead many researchers to identify other ILs that have a better solubility for biomass.

Recently, we showed that 1-allyl-3-methylimidazolium formate ([AMIM][HCOO]) dissolves 40% more hybrid poplar (HP) biomass than [EMIM][CH_3_COO] under identical dissolution conditions. Molecular dynamic simulations partially attributed this increased dissolution of biomass in [AMIM][HCOO] to stronger interactions with hemicellulose [37]. These results suggest that, during activation, [AMIM][HCOO] has enhanced interactions with each lignocellulosic component without the need to remove hemicellulose, as is believed to occur when using [EMIM][CH_3_COO]. To test this hypothesis, herein, we will evaluate and compare the commonly used IL [EMIM][CH_3_COO] versus [AMIM][HCOO] for effective biomass activation. More specifically, we investigated the physical and chemical features of HP biomass that arose due to IL activation, such as deacetylation, cellulose crystallinity, chemical composition, and anatomical characteristics. Statistical analyses were performed to analyze the chemical signature of the activated and regenerated biomass using Fourier transform infrared (FTIR) spectroscopy. Essentially, we will identify if [AMIM][HCOO] is an alternative to the commonly used [EMIM][CH_3_COO] in facilitating the production of sugars from biomass. Finally, while this work does not investigate the recovery and recycling of ILs, we recognize it is an essential component of any process that uses IL. Over the past few years, the IL community has been very active in addressing this challenge and several approaches have been recently proposed to accomplish this task [38–40].

## Methods

### Materials: biomass and ionic liquids

The biomass in this study, hybrid poplar (*Populus* spp.) (HP) wood, was obtained from the Center for Renewable Carbon at the University of Tennessee. Upon air-drying, the material was milled using a Wiley mill (Thomas Scientific™, Model # 3383-L10, Swedesboro, NJ) through a 40-mesh screen (0.425 mm). The HP powder was extracted in an Accelerated Solvent Extractor (ASE 350, Dionex, Sunnyvale, CA) to remove non-structural components [41]. Approximately 7 g of HP powder were mixed with 40 g of glass beads (3 mm) and added to a 66-mL extraction cell. Sequential extractions with water and ethanol were carried out at 10.3 MPa and 100 °C, with a 7-min static time per cycle (3 cycles). The wet, extractives-free HP material was then oven-dried at 40 °C until constant moisture content was reached (< 7% by weight). This extractive-free wood material was used throughout this study.

The ionic liquids, 1-ethyl-3-methylimidazolium acetate ([EMIM][CH_3_COO], purum ≥ 95%) and 1-allyl-3-methylimidazolium formate ([AMIM][HCOO], purum ≥ 95), were purchased from Iolitec Inc. (Tuscaloosa, AL) and used as received. Deionized water was used throughout the study.

### Activation and regeneration of HP in ionic liquids

The extractive-free HP material was activated using [EMIM][CH_3_COO] or [AMIM][HCOO] at a 10 wt% biomass loading. First, the ILs were weighed into a flask and heated to 100 °C to remove moisture. After 15 min, the temperature was set to 60 °C and the biomass was slowly added to the solvent. The biomass–IL mixture was agitated by a mechanical stirrer at 100 RPM for various time scales (3, 24, 48, and 72 h). After the respective periods, the biomass was regenerated by adding the same weight of deionized water as an anti-solvent and mixed for five additional minutes. The regenerated sample was recovered through seven rounds of water washing and vacuum filtration, using the same weight of water in each round as the weight of the IL–biomass mixture, and then dried in a 40 °C oven for 5 days.

A minimum of three replications were performed for each experimental condition and the recovered weight of biomass on dry basis was recorded. The complete removal of ILs from the biomass was confirmed by Fourier transform infrared (FTIR) spectroscopy and pyrolysis gas chromatography/mass spectrometry (Py-GC/MS).

### Chemical composition analysis

The chemical composition of the untreated and activated/regenerated HP material was determined based on procedures from NREL/TP-510-42618 [42]. The acid soluble lignin (ASL) content was measured at a wavelength of 240 nm using a Thermo Scientific™ GENESYS™ 10S UV–Vis spectrophotometer and the acid insoluble lignin (AIL) gravimetrically after ashing.

### Fourier transform infrared (FTIR) spectroscopy

The chemical signature of the HP samples was collected using a Perkin Elmer Spectrum One FTIR spectrometer (Waltham, MA). A small amount of biomass (~ 5 mg) was placed on an attenuated total reflectance (ATR) accessory of the spectrometer. FTIR spectra were collected over a range of 4000–600 cm^−1^ in absorbance mode, with a 4 cm^−1^ resolution and 8 scans per sample. Five spectra were collected for each sample. The spectra were pre-treated with an ATR correction, normalized, and corrected by Multiplicative Scatter Correction (MSC) in The *Unscrambler*^®^ X software version 9 (CAMO software).

### Statistical analysis: principal component analysis of FTIR spectra

Principal component analysis (PCA), a method of multivariate analysis, was used to analyze the FTIR spectral data. PCA allows for the visualization of composite data by identifying the main sources of variation and removing variability due to noise from the data. The spectral data are compressed and transformed into a data set that shows its most relevant factors, known as principal components (PCs). The first principal component (PC1) has the largest possible variance and accounts for most of the variation in the spectral data. The second principal component (PC2) then accounts for the second largest variance in the data, and so on. Scatter plots of principal component scores show the pattern of the data and are called scores plots. The relationship between wavenumber of the FTIR spectrum (variables) and the PCs is shown in a plot called loadings’ plot. The intensity of the variables in the loadings plot then shows how much each variable contributed to each PC [43].

### X-ray diffraction (XRD) of activated biomass

Following rapid screening by FTIR spectroscopy, the untreated and activated/regenerated HP samples were analyzed using powder X-ray diffraction for accurately determining the crystallinity of cellulose. The samples were individually mounted on a low-background quartz holder and measured using a PANalytical Empyrean X-ray diffractometer (PANalytical Inc., Westborough, MA), with a Cu–K-alpha tube (*λ* = 1.5418 Å) operating at a voltage of 45 kV and a current of 40 mA. The scatter angle, 2θ, was measured at a range of 9–41°, with a step size of 0.01°, using a 1/8° fixed divergence, a 1/4° anti-scatter slit, as well as a 0.04 rad Soller slit. The index of crystallinity (CrI) was determined using the Segal’s peak height method, as shown in the following equation:

1 $${\text{CrI = }}I_{002} - {{I_{\text{AM}} } \mathord{\left/ {\vphantom {{I_{\text{AM}} } {I_{002} }}} \right. \kern-0pt} {I_{002} }},$$

where *I*_002_ is the total intensity of the peak at 2*θ* = 22.5° and *I*_AM_ is the intensity of the background scatter at 2*θ* = 18.7° [44]. The CrI for cellulose was normalized against commercial Avicel, which was assigned a CrI of 100%. The XRD data were plotted and analyzed using the Origin 2017 SR 1 software (OriginLab Corporation).

### Anatomical characterization

Untreated and IL-activated/regenerated biomass samples were also characterized using scanning electron microscopy (SEM). A PhenomPro X desktop Scanning Electron Microscope was used to collect micrograph images of the samples at 50 kV using 400 and 1500× magnification. A total of ten images were taken for each sample and representative images are shown in this paper.

### Enzymatic saccharification

Following activation and regeneration, the HP samples were hydrolyzed with commercial enzymes. A biomass loading (BL) of 5 w/w% was used for saccharification with CTec3 cellulases and HTec3 hemicellulases (Novozymes). The saccharification was performed in triplicates, at a temperature of 50 °C in a 50-mM citrate buffer (pH 5.0), using capped Erlenmeyer flasks. The shaker was set to 100 RPM. Aliquots of the saccharified samples were taken at predetermined times of 0, 1, 3, 6, 12, 24, 48, and 72 h, boiled for 5 min to denature the enzymes, and centrifuged at 10,000 RPM (14,087×*g*) for 5 min. The aliquots were then filtered through 0.45-μm nylon membrane filters from Milli-pore (Billerica, MA) and analyzed by High-Performance Liquid Chromatography (HPLC). A Bio-Rad Aminex HPX-87P carbohydrate analysis column (Richmond, CA) and a deashing guard column (Bio-Rad, Hercules, CA) were used at 85 °C, with a mobile-phase (H_2_O) flow rate of 0.25 mL/min. To determine the acetyl content, a Bio-Rad Aminex HPX-87H column and a cation H guard column were used with a mobile-phase (H_2_SO_4_) flow rate of 0.6 mL/min.

### Results and discussion

The activation step performed in this work parallels the first 3 h of a typical dissolution process, when biomass is continuously stirred with the IL under controlled mixing and temperature conditions, which can last up to a week [37]. Unlike dissolution processes, activation results in biomass only partially dissolved in the IL; changes that take place during this phase can be studied by regenerating or precipitating the biomass after a short activation period through rapid addition of an anti-solvent. To assess these changes in our study, the following properties were measured: biomass regenerated yield, chemical composition, cellulose crystallinity, anatomical features, and ability of the regenerated biomass to enzymatically release sugars. The mass of regenerated biomass after IL activation is often a first indicator of the lignocellulosic component loss that takes place during activation. The data in Table 1 show the mass recovery for all the HP samples that were activated with [EMIM][CH_3_COO] and [AMIM][HCOO] at 10 wt% biomass loading for different periods.

**Table 1:** Mass recovery of hybrid poplar biomass (HP %) after activation with two ionic liquid types under varying times

Activation time (h)	Biomass mass recovery^a^ (% mean ± SD)	
	[EMIM][CH_3_COO]	[AMIM][HCOO]
3	96.1 ± 0.2	98.2 ± 0.0
24	96.1 ± 0.2	96.8 ± 0.4
48	96.1 ± 0.5	93.2 ± 1.0
72	94.1 ± 1.0	92.1 ± 0.8

^a^SD or standard deviations for mass recovery were calculated based on triplicates

The mass of recovered IL-activated HP biomass decreases with increasing activation time, with a maximum loss of ~ 6 and 8% during the 72-h activation period for [EMIM][CH_3_COO] and [AMIM][HCOO], respectively. This loss is not attributed to the removal of water, extractives, or inorganics from biomass, as these are accounted for during the experiment and previously extracted before activation. For [EMIM][CH_3_COO]-activated biomass, the mass loss has been attributed to the deacetylation of hemicellulose and lignin during the activation [25, 27]. Such deacetylation reactions have not yet been documented using [AMIM][HCOO].

**Fig. 1:**
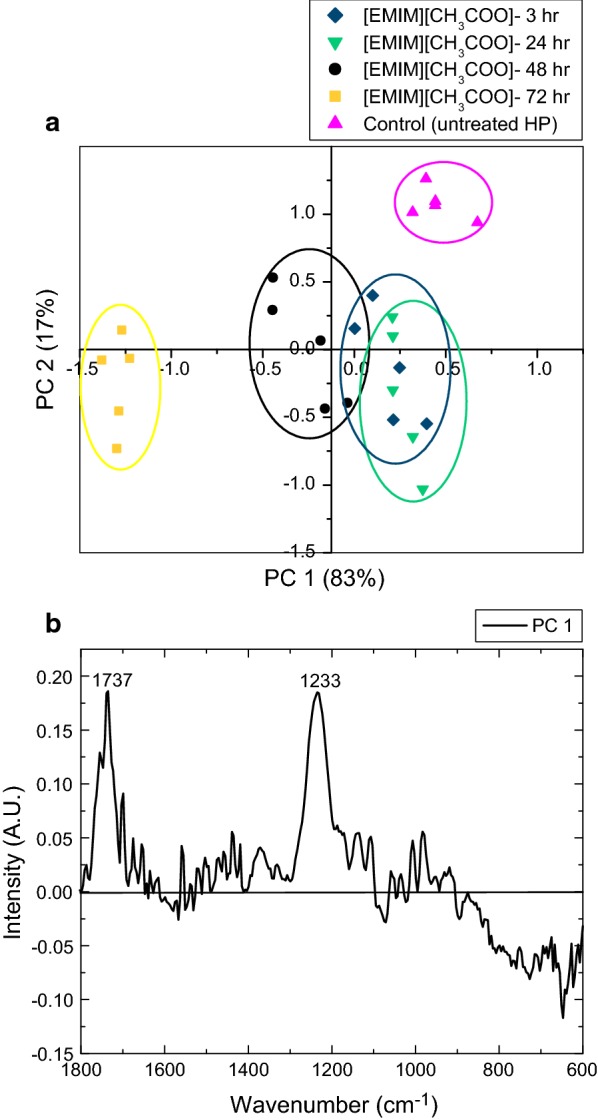
Principal component analysis (PCA) on [EMIM][CH_3_COO]-activated samples compared to untreated hybrid poplar. The principal component analysis (PCA) for [EMIM][CH_3_COO]-activated samples at 3-, 24-, 48-, and 72-h activation times compared to an untreated control sample. The scores plot is shown on the top (**a**), and the loadings’ plot for PC1 on the bottom (**b**)

**Fig. 2:**
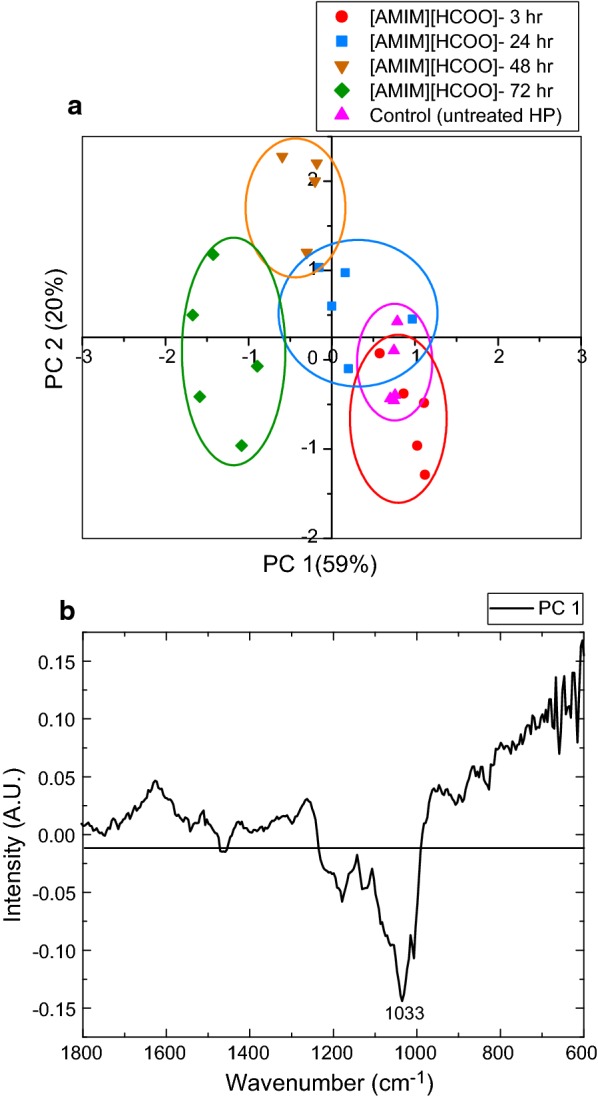
Principal component analysis (PCA) on [AMIM][HCOO]-activated samples compared to untreated hybrid poplar. The principal component analysis (PCA) for [AMIM][HCOO]-activated samples at 3-, 24-, 48-, and 72-h activation times compared to an untreated control sample. The scores plot is shown on the top (**a**), and the loadings’ plot for PC1 on the bottom (**b**)

To verify these changes, an initial screening of the chemical signature of the IL-activated HP biomass was performed using FTIR spectroscopy coupled with principal component analysis (PCA). Figures 1 and 2 present scatter plots of principal component scores, i.e., scores’ plots, showing the pattern in the FTIR data, and a loadings plot displaying the relationship between the wavenumbers of the FTIR spectrum and the PCs. PCA scores and loadings plots highlight the significant differences caused by the diverse activation times for [EMIM][CH_3_COO] (Fig. 1a, b, corresponding to scores and loadings plot, respectively) and [AMIM][HCOO] (Fig. 2a, b, corresponding to scores and loadings plot, respectively) when compared to the control that consisted of untreated HP in the FTIR fingerprint region. For [EMIM][CH_3_COO]-activated HP biomass, the scores plot shows that samples activated for 3, 24, and 48-h clustered along PC1 with the 72-h activated samples being the farthest away from the untreated biomass (control). The PC1 loadings plot highlights that significant spectral changes occurred at 1737 and 1233 cm^−1^; both bands assigned to acetyl group vibrations, C=O and C–O stretch, respectively [45, 46]. Since the acetyl group bands are positive, as shown in Fig. 1b, and the 72-h [EMIM][CH_3_COO]-activated samples are located in the negative quadrant of PC1, as shown in Fig. 1a, it infers that there are fewer acetyl groups in these 72-h samples, as compared to the control and samples that were treated with shorter activation times [27].

Similarly, the scores’ plot for the [AMIM][HCOO]-activated HP samples at different times is shown in Fig. 2a, with the 72-h activated samples being the furthest from the control. However, unlike with [EMIM][CH_3_COO], the PC1 loadings plot for [AMIM][HCOO]-activated samples (Fig. 2b) does not display a significant difference in the acetyl region. Nevertheless, there is an intense negative band at 1033 cm^−1^ which is associated with C–O and C–C–O functional group stretching, indicating changes in biomass activated with [AMIM][HCOO] for 72 h compared to the other samples. This observation and the data presented in Table 1 indicate that the biomass loss observed during activation with [AMIM][HCOO] is not primarily due to deacetylation like with [EMIM][CH_3_COO], but could be attributed to the loss of some carbohydrates (C–O stretching in cellulose, and hemicellulose), and subsequent biomass removal during the washing step [47, 48]. Overall, similar sample clustering for both ILs demonstrates that longer activation times have a greater impact on the chemical features of HP biomass. However, unlike [EMIM][CH_3_COO], deacetylation is not the biggest change observed when HP is activated with [AMIM][HCOO].

**Fig. 3:**
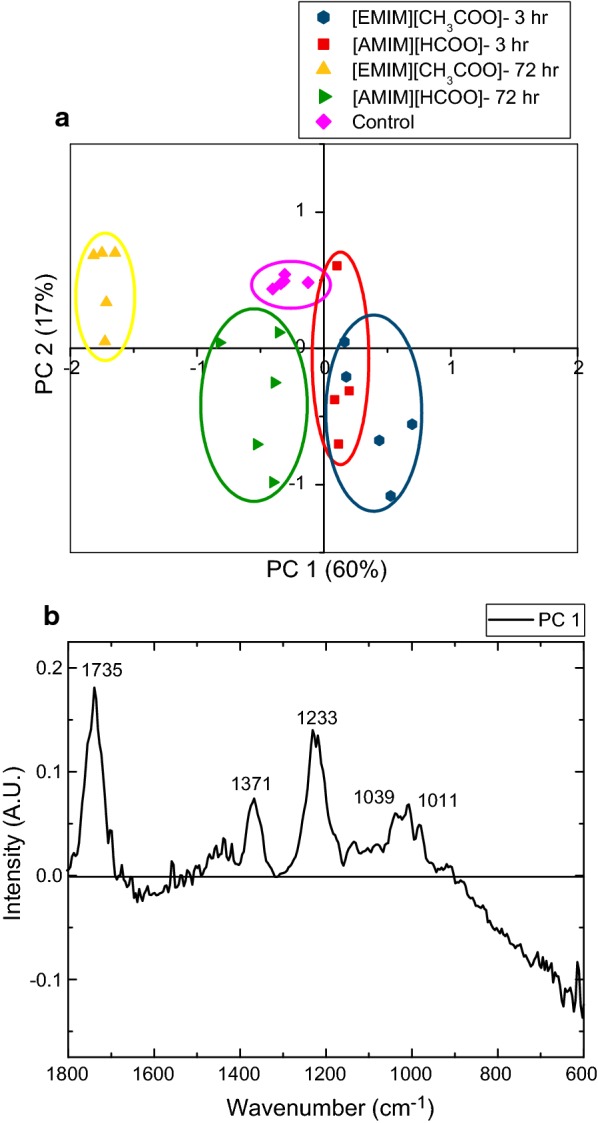
Principal component analysis (PCA) on selected [EMIM][CH_3_COO] and [AMIM][HCOO]-activated hybrid poplar. Principal component analysis (PCA) scores’ plot of IL-activated hybrid poplar compared to untreated HP control (**a**). PCA loadings plot for PC1 (**b**)

To streamline our efforts and provide direct comparison to previous studies, we focused our subsequent analyses only on the shortest (3 h) and the longest (72-h) activation times for each IL [22, 49, 50]. In addition, these activation times were selected on the basis that 72-h activated hybrid poplar biomass can enzymatically produce the same amount of glucose as samples activated for 3 h coupled with autohydrolysis [35]. PCA of the FTIR spectra collected for the 3- and 72-h activated samples of both ILs was performed to investigate the chemical features of these samples by IL type and activation time (Fig. 3a, b). According to the loadings’ plot (Fig. 3b), the most significant spectral changes occur at 1735, 1371, 1233, 1039, and 1011 cm^−1,^ with 1735, 1371, and 1233 cm^−1^ having the highest intensities. The two most intense bands at 1735 and 1233 cm^−1^ are assigned to acetyl group vibrations, whereas the band at 1371 cm^−1^ is attributed to C–H deformation in cellulose and hemicellulose [47, 48]. Similar to the observation for Fig. 1, the acetyl group bands in Fig. 3b are positive and the 72-h activated samples for both ILs are located on the negative quadrant of PC1. However, the 72-h [EMIM][CH_3_COO]-activated samples are located further away from the rest of the samples projected onto the PCs axes. Overall, variations in the FTIR spectra confirm that [EMIM][CH_3_COO] altered the chemical features of biomass the most, while the use of [AMIM][HCOO] does not seem to significantly impact the chemical fingerprint of the resulting biomass. This observation was clearly seen, whereby the 72-h [AMIM][HCOO]-activated biomass is located near the control and 3-h [AMIM][HCOO], indicating similar chemical features.

To confirm the FTIR findings and investigate if any specific biomass constituent was hydrolyzed/lost during activation, the chemical composition of the ionic liquid activated/regenerated HP samples was determined. Table 2 corroborates that the regenerated samples have lower acetyl content than the starting material (control). After a 3-h activation period, acetyl content decreases from 5.9% in the control sample to 5.3% and 4.9% in [EMIM][CH_3_COO] and [AMIM][HCOO] regenerated samples, respectively. After 72-h activation with [EMIM][CH_3_COO], acetyl content drops to 3.3% which corresponds to a reduction of 44.1% compared to the control. Acetyl content for [AMIM][HCOO]-treated samples with the same 72-h activation time shows a much smaller decrease (from 5.9 to 4.8%; a decrease of 18.6%). Contrary to [EMIM][CH_3_COO], these results indicate that only a small fraction of the acetyl groups is lost during activation with [AMIM][HCOO].

**Table 2:** Chemical composition (% dry basis) of regenerated hybrid poplar biomass after [EMIM][CH_3_COO] and [AMIM][HCOO] activation at 10 wt% loading for varying activation times

Chemical composition	Control HP (untreated)	[EMIM][CH_3_COO]-activated HP		[AMIM][HCOO]-activated HP	
Activation time (h)	–	3	72	3	72
Cellulose (%)	44.2^a^ ± 0.2	43.4^b^ ± 0.1	43.5^b^ ± 0.3	42.8^c^ ± 0.2	42.6^c^ ± 0.1
Hemicellulose (%)	20.6^a^ ± 0.1	20.2^a^ ± 0.1	19.9^a^ ± 0.2	19.0^b^ ± 0.2	18.5^b^ ± 0.3
Total carbohydrates (%)^1^	64.5^a^ ± 0.9	63.7^ab^ ± 0.3	63.4^b^ ± 0.5	61.8^c^ ± 0.3	61.2^c^ ± 0.2
Lignin (%)	27.8^b^ ± 0.6	28.9^a^ ± 0.2	27.8^b^ ± 0.3	27.9^b^ ± 0.3	27.5^b^ ± 0.5
Acetyl content (%)	5.9^a^ ± 0.07	5.3^b^ ± 0.05	3.3^e^ ± 0.05	4.9^c^ ± 0.02	4.8^d^ ± 0.03
Decrease in acetyl content (%)^2^	–	10.2	44.1	16.9	18.6

Mean ± standard deviation are based on triplicate measurements

Least significant difference (LSD) test was performed to determine differences within each biomass constituent. Means with the same letter (a, b, c, d, and e) are not significantly different by LSD at *p* < 0.05

^1^Total carbohydrate content is the sum of cellulose and hemicellulose

^2^${\text{Decrease in acetyl content}} = \frac{{ {\text{Acetyl}}_{\text{Control}} - {\text{Acetyl}}_{{{\text{IL}} - {\text{activated}} {\text{HP}}}} }}{{{\text{Acetyl}}_{\text{Control}} }} \times 100\% ,$ with IL being [EMIM][CH_3_COO] or [AMIM][HCOO]

**Fig. 4:**
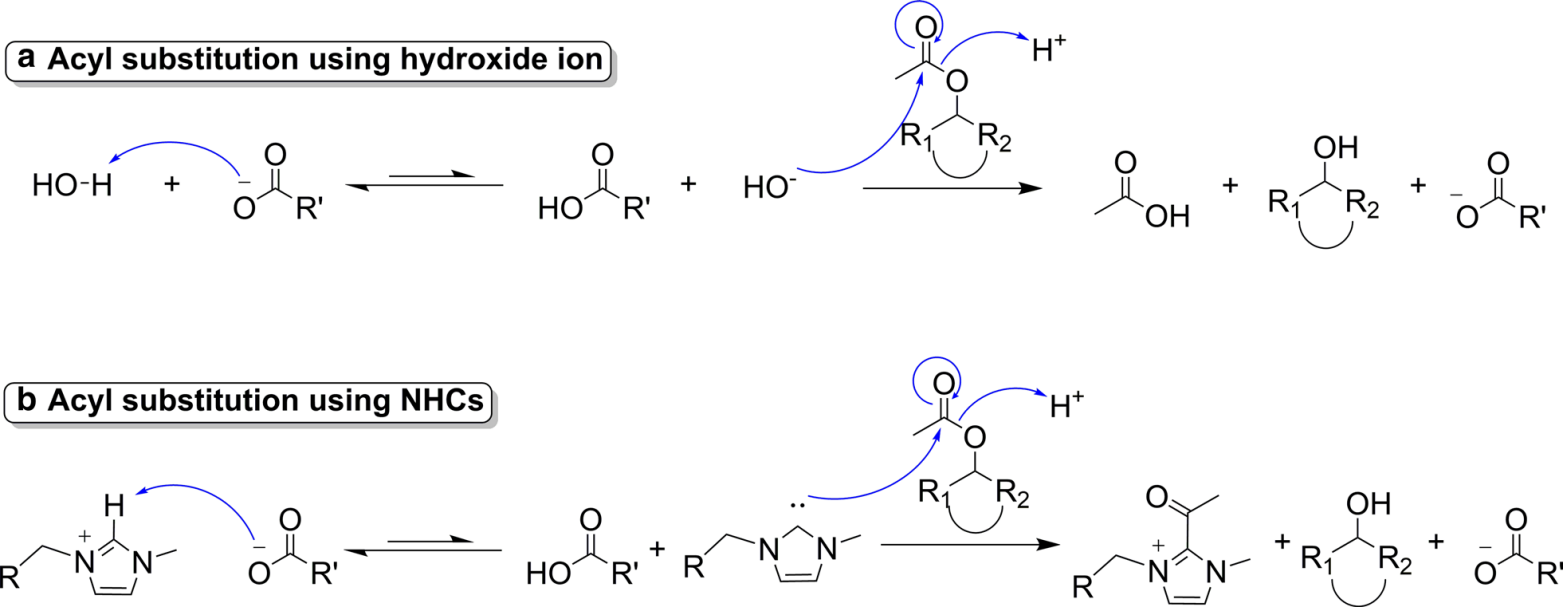
Mechanism for deacetylation of biomass using ionic liquids. In the mechanism for proposed biomass deacetylation using [EMIM][CH_3_COO] or [AMIM][HCOO], R represents the H or CH_3_ attached to the formate or acetate anion. R_1_ and R_2_ represent the carbon chain on the biomass structure

When testing each IL, all hybrid poplar biomass activations were conducted under identical conditions. Because of this, we propose that the larger percentage of acetyl group cleavage observed when using [EMIM][CH_3_COO] is unlikely a result of simple thermal hydrolysis by adventitious water. Likewise, the numerous structural similarities of [EMIM][CH_3_COO] and [AMIM][HCOO] suggest that they should undergo relatively similar chemistries, to a first approximation, if they are, indeed, active participants in the observed deacetylation chemistry. Therefore, we hypothesize that the observed differentiation may stem from basicity differences in the IL’s respective anions, acetate and formate. More specifically, the pKa of formic acid (the conjugate acid of formate) is 3.75 and is lower than that of acetic acid (the conjugate acid of acetate) which has a pKa value of 4.76. Because of this pKa difference, we can safely surmise that acetate is a stronger base than formate and can, therefore, produce a one order of magnitude higher equilibrium concentration of nucleophilic species than when using formate-based ILs. Such nucleophilic species may include hydroxide, which results upon deprotonation of water (Fig. 4a), or *N*-heterocyclic carbenes (NHCs), which result upon deprotonation of the imidazolium cations (Fig. 4b) [51], and both of which are known to readily attack acetyl esters. Because acetate is the stronger base and should result in a higher concentration of these nucleophilic species, we hypothesize that this may promote biomass deacetylation to a greater extent, as was observed when using [EMIM][CH_3_COO] [52].

Interestingly, further analysis of the data in Table 2 reveals a reduction in the carbohydrates content for [AMIM][HCOO]-activated biomass compared to the control (untreated HP). After 72-h activation in [AMIM][HCOO], the carbohydrates content in the regenerated biomass (61.2%) is lower by 5.1% compared to the untreated biomass (64.5%), while lignin content remains constant. These findings agree well with the FTIR results and could justify the lower mass recovery of 92.1% for the [AMIM][HCOO]-activated biomass.

**Fig. 5:**
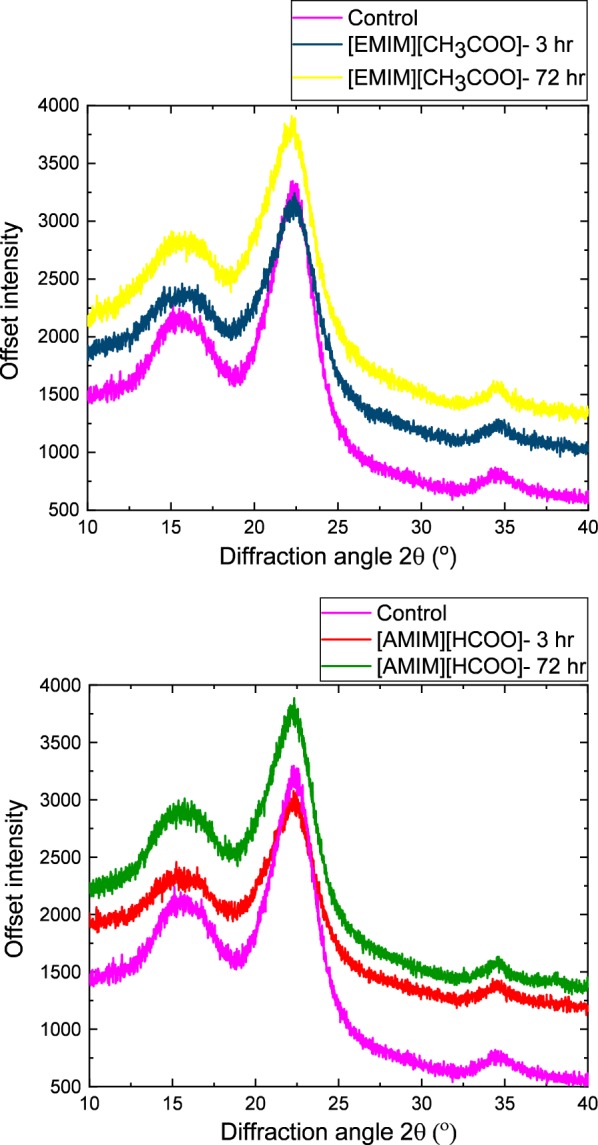
Cellulose crystallinity of IL-activated samples at 3 and 72 h compared to untreated hybrid poplar. X-ray diffraction (XRD) patterns for IL-activated sample at 10 wt% biomass loading are compared to the untreated control. Index of crystallinity for cellulose was calculated using Eq. 1

In addition to chemical changes, the crystallinity of the activated/regenerated biomass was investigated by X-ray diffraction (Fig. 5). Activation with both ILs slightly modifies the crystalline profile of the biomass with a slight broadening of the main peak at 2*θ* = 22.5° compared to the control. Although no peak shifts are observed, which are usually indicative of cellulose I transitioning into cellulose II during dissolution, there is a slight decrease in peak intensity at 35° for the 72-h activated samples. We hypothesize that this change is due to a possible disruption of the microfibril alignment of the cellulose chains [53]. An index of crystallinity (CrI) calculated from the XRD patterns is provided in Table 3. Similar to acetyl content in Table 2, a small decrease in the CrI for the 3-h activated samples is observed, with a 17.6% and 11.7% decrease for [EMIM][CH_3_COO] and [AMIM][HCOO], respectively, compared to the control. However, for the 72-h activated samples, [EMIM][CH_3_COO] reduces cellulose crystallinity of hybrid poplar biomass from 61.4 to 43.5%, while the cellulose crystallinity for [AMIM][HCOO] only drops to 49.6%. Overall, when comparing the two ILs and activation times, the data confirm a higher structural disruption of the biomass primary components with [EMIM][CH_3_COO] at 72 h. Literature shows that, as this solvent penetrates into the hydrogen bonded sheets of cellulose I, the lattice structure expands and causes biomass to undergo crystallinity changes [54].

**Table 3:** Comparison of index of crystallinity (CrI) measured through XRD for [EMIM][CH_3_COO] and [AMIM][HCOO]-activated hybrid poplar samples

	Control HP	[EMIM][CH_3_COO]-HP		[AMIM][HCOO]-HP	
Activation time (h)	–	3	72	3	72
Index of crystallinity (CrI %)	61.4	50.6	43.5	54.2	49.6
Decrease in crystallinity (%)^a^	–	17.6	29.2	11.7	19.2

^a^${\text{Decrease in crystallinity}} = \frac{{ {\text{CrI}}_{\text{Control}} - {\text{CrI}}_{{{\text{IL}} - {\text{HP}}}} }}{{ {\text{CrI}}_{\text{Control}} }}$, with IL being [EMIM][CH3COO] or [AMIM][HCOO]

**Fig. 6:**
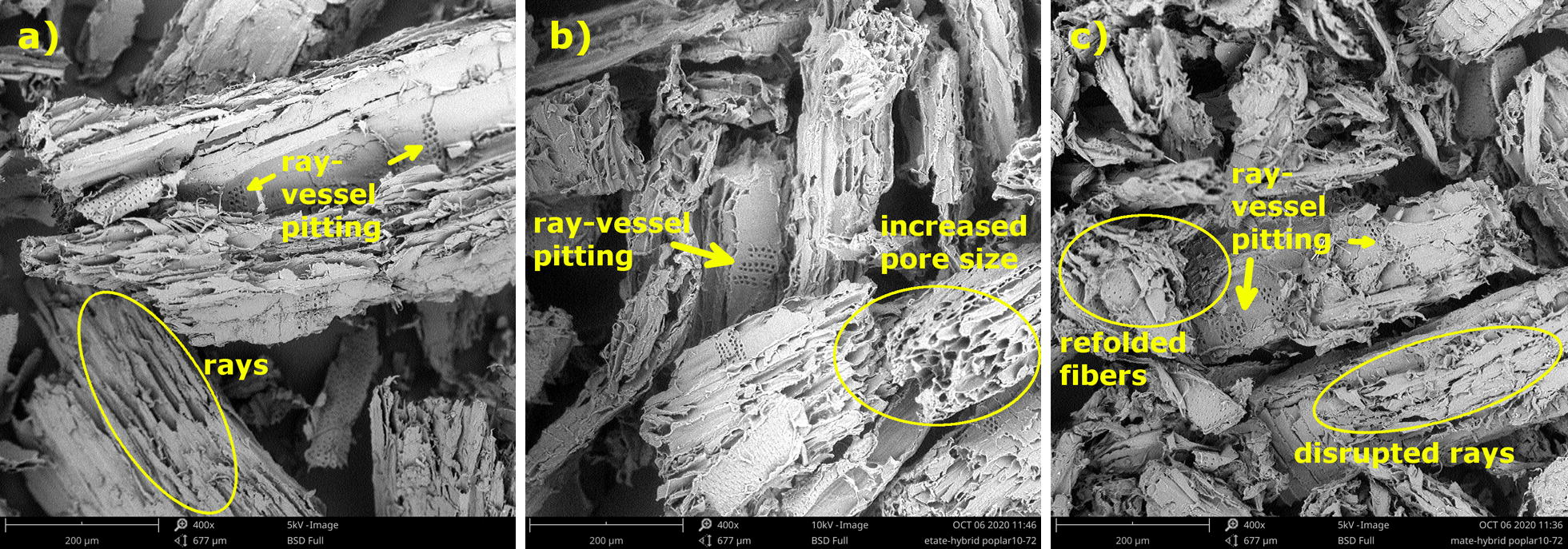
Scanning electron microscopy (SEM) images for untreated hybrid poplar and IL-activated samples after 72 h. The SEM micrographs of control (untreated HP) (**a**), [EMIM][CH_3_COO]-activated HP (**b**), and [AMIM][HCOO]-activated HP (**c**). Images were taken at ×400 magnification

To visualize potential physical and anatomical changes that took place during the 72-h IL activation, scanning electron microscopy (SEM) images were collected (Fig. 6). The features of hardwood tissues are clearly observed in the control (untreated HP), with visible ray-vessels’ pittings, fibers, and ray cells [55]. The morphology and structural ordering of [EMIM][CH_3_COO] and [AMIM][HCOO]-activated biomass appear to have subtle differences compared to each other and to the control. One similarity for both IL treatments is the lack of lignin droplet accumulation on the cellulose fibers, indicating that the activation step was mild and did not significantly impact the anatomical features of the biomass [56]. Previous studies using dilute acid pretreatment have reported the coalescence of lignin droplets on the surface of wood, presenting a barrier for enzymatic hydrolysis [57]. Moreover, the regenerated biomass samples in this study do not show unpacking of macro fiber bundles or loss of structural ordering of biomass, which occur during pretreatments with higher severity, as reported by Singh et al. [22].

However, subtle differences could be observed when comparing the IL samples and the control. The [EMIM][CH_3_COO]-activated biomass (Fig. 6b) appears to have larger ring-like porous structures around the microfibrils. Rays are still seen and the activation seemed to have somewhat changed the structure of the wood making it more porous, which will make the cellulosic component of the biomass more accessible for further degradation using enzymes. On the other hand, the SEM images for [AMIM][HCOO]-activated biomass (Fig. 6c) seem to show re-folding of the fibers. It appears if the activation only disrupted the ray cells of the biomass, without affecting its porosity. Overall, these slight differences demonstrate that activation with these two ionic liquids under mild conditions (short time and temperature < 100 °C) does not significantly impact the physical and anatomical features of lignocellulosic biomass.

**Fig. 7:**
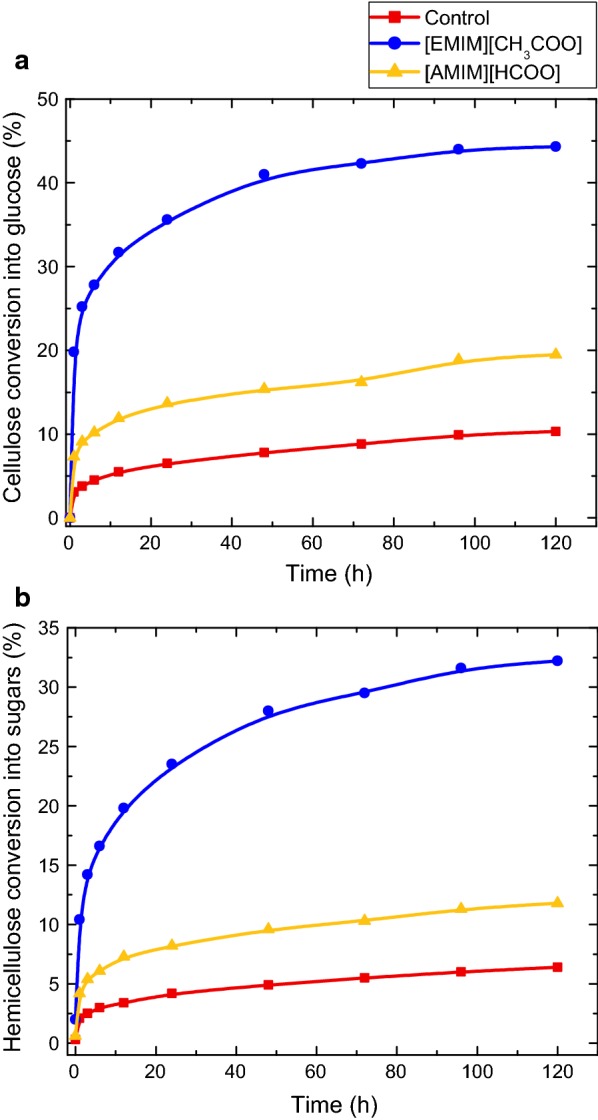
Enzymatic saccharification on cellulose and hemicellulose components of IL-activated samples. The kinetics of enzymatic saccharification on cellulose (**a**) and hemicellulose (**b**) of 72-h activated biomass at a 10 wt% biomass loading are shown in this figure. The conversion was calculated based on the chemical composition of raw hybrid poplar

The chemical and physical features of biomass are correlated to the enzymatic hydrolysis of cellulose and hemicellulose. Using commercial enzymes, the enzymatic saccharification of cellulose and hemicellulose for the 72-h [EMIM][CH_3_COO] and [AMIM][HCOO]-activated samples was monitored and presented in Fig. 7a, b, respectively. The highest cellulose conversion of 44% is obtained for the [EMIM][CH_3_COO]-activated sample after a 72-h activation, while only 20% of the cellulose in the [AMIM][HCOO]-activated sample are converted into soluble sugars. Similarly, the highest hemicellulose conversion is obtained for [EMIM][CH_3_COO] activation of the HP biomass. These observed conversion trends show similarities to the CrI trend in Table 3.

The observations from FTIR spectroscopy, X-ray diffraction, SEM, and enzymatic saccharification confirm that [EMIM][CH_3_COO] and [AMIM][HCOO] do not activate hybrid poplar biomass at the same extent. [EMIM][CH_3_COO] activation deacetylates hemicellulose to a higher degree and significantly decreases cellulose crystallinity. These occurrences are not as pronounced during activation with [AMIM][HCOO] despite a larger amount of biomass is dissolved in this IL [37]. Instead, the [AMIM][HCOO]-activated hybrid poplar seems to show folding of the fibers on its surface. In our case, this potential phenomenon could be due to additional hydrogen bonds formed (during activation) and preserved during the regeneration step with water (anti-solvent).

Nevertheless, the behavior of [AMIM][HCOO] during activation is a highly valued characteristic of ILs for the direct production of bioproducts such as films and fibers from whole biomass-IL system [36]. Since [AMIM][HCOO] is known to have better dissolution properties than [EMIM][CH_3_COO] [37] and the literature shows that the former has lower viscosity than the latter [37], there is potential for [AMIM][HCOO] to become a better solvent for fiber spinning. Although [EMIM][CH_3_COO] has been used for films casting in recent studies [36], our work shows that [EMIM][CH_3_COO] performs better at activating and thereby producing sugars and lignin through a fractionation approach, while [AMIM][HCOO] performs better at dissolving whole biomass for the direct processing of products. Particularly, this IL does not require the partial removal of hemicellulose to achieve higher biomass loading (which results in higher energy efficiency and cost-effectiveness of the process) and generate IL-biomass solution with adequate rheological properties, i.e., viscosity behavior and flow properties of the system, both important during handling, dissolution, and processing [58].

## Conclusions

Our results show that hybrid poplar biomass activation at 60 °C using both ILs acetate and formate impacted acetyl content and crystallinity, with the former IL having more of an impact than the latter. During hybrid poplar activation using [EMIM][CH_3_COO] for 72 h, the biomass undergoes a deacetylation of 44.1%, has cellulose crystallinity reduced by 29.3%, and results in 44% cellulose conversion during enzymatic saccharification. The uncommon IL [AMIM][HCOO] did not produce similar results, retaining 81.4% of the acetyl groups in the biomass, and decreasing the crystallinity of cellulose by only 19.2% even after 72-h mixing under 60 °C. These findings highlight the potential for new research opportunities for using [AMIM][HCOO] to dissolve biomass and direct processing into fibers or other materials with enhanced properties.

## Abbreviations

IL: ionic liquid
HP: hybrid poplar
BL: biomass loading
[EMIM]: 1-ethyl-3-methylimidazolium
[AMIM]: 1-allyl-3-methylimidazolium
[CH_3_COO]: acetate
[HCOO]: formate
FTIR: Fourier-transform infrared spectroscopy
PCA: principal component analysis
XRD: X-ray diffraction
CrI: crystallinity index
SEM: scanning electron microscopy
